# An Adaptive Ship Detection Scheme for Spaceborne SAR Imagery

**DOI:** 10.3390/s16091345

**Published:** 2016-08-23

**Authors:** Xiangguang Leng, Kefeng Ji, Shilin Zhou, Xiangwei Xing, Huanxin Zou

**Affiliations:** School of Electronic Science and Engineering, National University of Defense Technology, Sanyi Avenue, Changsha 410073, China; luckight@163.com (X.L.); slzhou@nudt.edu.cn (S.Z.); xingxiangwei@nudt.edu.cn (X.X.); hxzou2008@163.com (H.Z.)

**Keywords:** adaptive ship detection, ship candidate detection, ship discrimination, constant false alarm rate (CFAR), confidence probability, synthetic aperture radar (SAR)

## Abstract

With the rapid development of spaceborne synthetic aperture radar (SAR) and the increasing need of ship detection, research on adaptive ship detection in spaceborne SAR imagery is of great importance. Focusing on practical problems of ship detection, this paper presents a highly adaptive ship detection scheme for spaceborne SAR imagery. It is able to process a wide range of sensors, imaging modes and resolutions. Two main stages are identified in this paper, namely: ship candidate detection and ship discrimination. Firstly, this paper proposes an adaptive land masking method using ship size and pixel size. Secondly, taking into account the imaging mode, incidence angle, and polarization channel of SAR imagery, it implements adaptive ship candidate detection in spaceborne SAR imagery by applying different strategies to different resolution SAR images. Finally, aiming at different types of typical false alarms, this paper proposes a comprehensive ship discrimination method in spaceborne SAR imagery based on confidence level and complexity analysis. Experimental results based on RADARSAT-1, RADARSAT-2, TerraSAR-X, RS-1, and RS-3 images demonstrate that the adaptive scheme proposed in this paper is able to detect ship targets in a fast, efficient and robust way.

## 1. Introduction

Synthetic Aperture Radar (SAR) is a microwave remote sensing technology capable of providing 2D images with fine resolutions and 24-h all-weather sensing capability [1]. Moreover, spaceborne SAR provides large scenes and keeps working for a long time. Therefore, ship detection is in a unique position to benefit from spaceborne SAR [2,3,4]. Ship detection in spaceborne SAR imagery has many applications, including ship traffic monitoring, sustainable economic development and environmental protection [5,6,7].

Since the launch of the Seasat in 1978, the number of spaceborne SAR sensors has drastically increased [5]. Table 1 summarizes current typical on-orbit SAR sensors. Besides, more than 10 additional SAR sensors will be launched over the next 10 years [8]. At the same time, several organizations have developed a number of SAR ship detection systems, such as OMW (Ocean Monitoring Workstation) [9], IMPAST (Improving Fisheries Monitoring Through Integrating Passive and Active Satellite Based Technologies) [10], SIMONS (Ship Monitoring System) [11] and ShipSurveillance [12], etc. However, most of them are designed for early SAR sensors, such as ERS (European Remote Sensing satellites) and ENVISAT (ENVIronmental SATellite). Usually, they are tailored to specific sensors, imaging modes, or resolutions. New characteristics of current high resolution, wide swath, multi-frequency, multi-polarization, or multi imaging mode SAR sensors are more challenging. Recently, a number of systems can process images of the current satellites (e.g., Sentinel-1, RADARSAT-2, and ALOS-2 (Advanced Land Observing Satellite)), including multi-polarization, high and low resolution images [3,4,7,13,14]. Besides, ISR/IO (Intelligence, Surveillance, Reconnaissance, Information Operations) Department of SPAWAR (Space and Naval Warfare Systems Command) Systems Center Pacific [15] proposed an adaptive ship detection system. However, its detail is not clear enough. Another challenge of ship detection today is how to improve the coverage and revisit to meet the needs of continuous surveillance. Thus, RADARSAT Constellation Mission (RCM) [16] or similar concepts are essential. There are also other data sources used to detect ships, e.g., optical imagery [17], AIS (Automatic Identification System) [5,18], altimetry [19], etc. Thus, how to use SAR imagery in synergy with other data sets to get better coverage in space and time is also an important application scenario.

**Figure 1:**
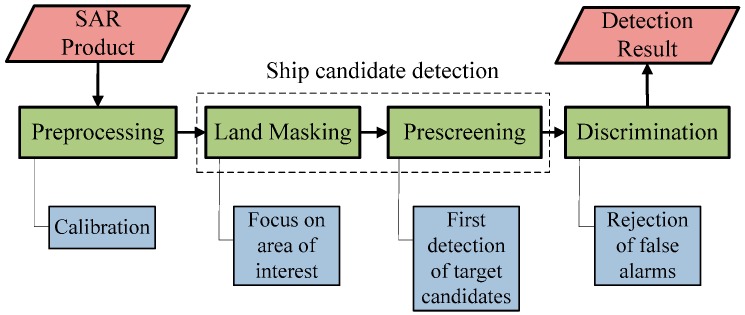
Overview of ship detection strategy.

Generally, ship detection systems consist of four stages [13,25] as shown in Figure 1: preprocessing, land masking, prescreening, and discrimination. The preprocessing stage includes radiometric and geometric correction usually. It makes the subsequent detection stages easier and more accurate. Then, both land masking and prescreening comprise the ship candidate detection stage. The function of this stage is to identity all potential ship targets, i.e., the ship candidates, in a fast and robust way. However, merely one algorithm or even a combination of algorithms would not do this perfectly. Consequently, there are false alarms in the preliminary detection result usually. Thus, the discrimination stage follows up. In the discrimination stage, false alarms are identified and rejected. Finally, the detection result is put forward after ship discrimination.

In this paper, a highly adaptive ship detection scheme for spaceborne SAR imagery is proposed. The scheme is composed of two stages: ship candidate detection and discrimination. The ship candidate detection includes land masking and prescreening. The main novelties in this study can be summarized as follows:

- This paper designs a complete ship detection scheme in a reliable and practical fashion. The scheme focuses on practical problems of adaptive ship detection, capable of processing a wide range of sensors, imaging modes and resolutions. Thus, a lot of practical problems, such as, how to decide the constant false alarm rate (CFAR) [2] window sizes in different resolution SAR imagery, and how to take advantage of SAR system metadata, ship size and pixel size in ship detection, are discussed in this paper.
- This paper applies two different detection strategies to high and low resolution SAR imagery, respectively. CFAR is the core of prescreening in this paper. By taking into account the imaging mode, incidence angle, and polarization mode of SAR imagery, it implements the adaptive ship candidate detection in spaceborne SAR imagery. Experimental results show that the adaptive ship candidate detection is able to detect ship targets in a fast, efficient and robust way.
- This paper proposes a comprehensive ship discrimination method based on confidence level and complexity. In practical applications, one sole discrimination method would not identify variable false alarms perfectly. Besides, automation process without eye inspection cannot always guarantee the correctness of discrimination results. The proposed comprehensive ship discrimination method is able to discriminate different types of typical false alarms.
- This paper categories discrimination based on features into certain discrimination (CeD) and confidence discrimination (CoD) based on confidence level. It then proposes to discriminate false alarms caused by azimuth ambiguities. It is able to combine automation process and manual work efficiently based on the confidence probability proposed in this paper. Each detection result is marked with a confidence probability instead of True/False.

The rest of this paper is organized as follows. Section 2 proposes an adaptive land masking method firstly, and then proposes an adaptive prescreening method. Section 3 proposes a comprehensive ship discrimination method. Section 4 validates the scheme proposed in this paper by testing on real SAR images from RADARSAT-1, RADARSAT-2, TerraSAR-X, RS-1, and RS-3 of China. Finally, Section 5 concludes this paper.

## 2. Ship Candidate Detection

In this section, we first propose an adaptive land masking method to remove land in spaceborne SAR imagery, and then propose an adaptive ship candidate detection method to detect all ship targets fast and robustly.

### 2.1. Land Masking

**Figure 2:**
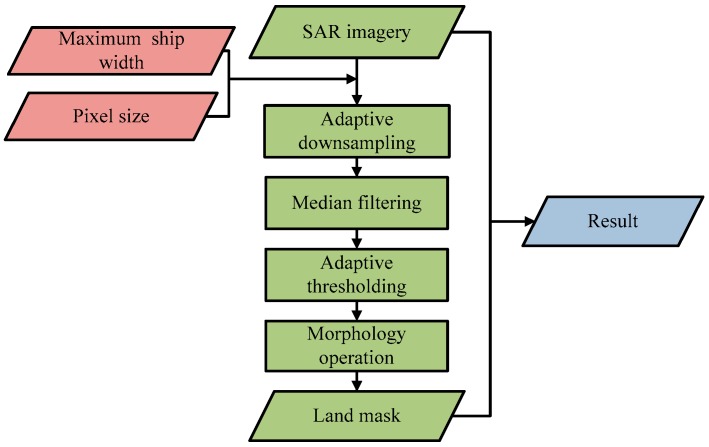
Detailed flow of land masking.

Since land areas are more complex than the sea, prescreening can produce many false alarms when applied to land areas. As only ships in the ocean are of interest, land masking is quite important. There are two common approaches for land masking [26]. One is to register the SAR image with existing geospatial databases [13], e.g., the GSHHS (Global Self-consistent, Hierarchical, High-resolution Shoreline) [27]. Another one is to use automatic algorithms to detect coastlines or land areas [28,29]. In this paper, an adaptive land masking method is proposed using ship size and pixel size. If several polarization channels are present, polarization channel used for land masking is based on per image. The decision to use which polarized channel will be elaborated in the next stage. Figure 2 shows its detailed flowchart, which can be divided into five steps.

#### 2.1.1. Downsampling the SAR Image Adaptively

As land areas and islands are big structures while ships are small structures, it is more suitable to use the downsampled image to analyze land masking according to the multiresolution analysis theory [30]. Besides, downsampled images will reduce the time consumption. It should be noted that before downsampling the image has been averaged. In this step, downsampling in spatial domain is applied, and downsampling rate R is set adaptively, R=shipwidth/pixelsize, where shipwidth is the maximum width of ship targets, pixelsize is the pixel size of the SAR image. Obviously, R is the pixel number of the maximum width of ship targets. The main effect of this step is to remove ship targets or to turn ship targets into isolated points. Contours of land areas and islands are kept because of their bigger structure. However, islands with a size below the maximum width of a ship will not be masked out by the algorithm. This can cause false alarms and should be improved in the future. Empirically, the maximum width of ship targets is approximately 60 m. Thus, if the maximum width is not known in SAR images, shipwidth will be set as 60 m.

#### 2.1.2. Median Filtering the Sampled Image

In this step, a median filtering with 3 × 3 window is scanned over the entire image. At each step in the scan, the center pixel is replaced by the median value of the 3 × 3 window. As ship targets are isolated points or a line in the downsampled image at most, median filtering with 3 × 3 window will wipe out all ship targets. At the same time, the whole image is smoothed while the transition points or edges will not be blurred. This is important when accurate operation is required.

#### 2.1.3. Thresholding the Filtered Image

In this step, we apply a distinction between land and water. Generally, the radar returns from land are expected to be higher than water. As a result, pixel values of land are higher than water. Otsu method [31] is employed to segment the image. The land mask value y(i,j) is calculated as follows,

(1) $$y\left(i,j\right)=\left\{ \begin{array}{l} 1,\;if\;x\left(i,j\right)\geq T\\ 0,\;if\;x\left(i,j\right)<T \end{array}\right..$$

where T is the threshold calculated by the Otsu method, 1 represents land areas while 0 water.

#### 2.1.4. Morphology Processing the Thresholded Image

Rivers and lakes are similar to the sea, which can generate shadow regions in the interior land. Similarly, land in a shadow can have a very low radar return, since it is not illuminated with much radar energy. Thus, morphology operation is important. In this step, we prefer the filling and dilation operation. Above all, the holes in the land mask should be filled. Then, the boundary of the land should be dilated to make a continuous boundary without any gap.

#### 2.1.5. Mapping the Land Mask to Get the Result

In this step, the land mask is mapped to the original SAR images. Land areas are marked as 1 (white), and the water is marked as 0 (black).

### 2.2. Prescreening

**Figure 3:**
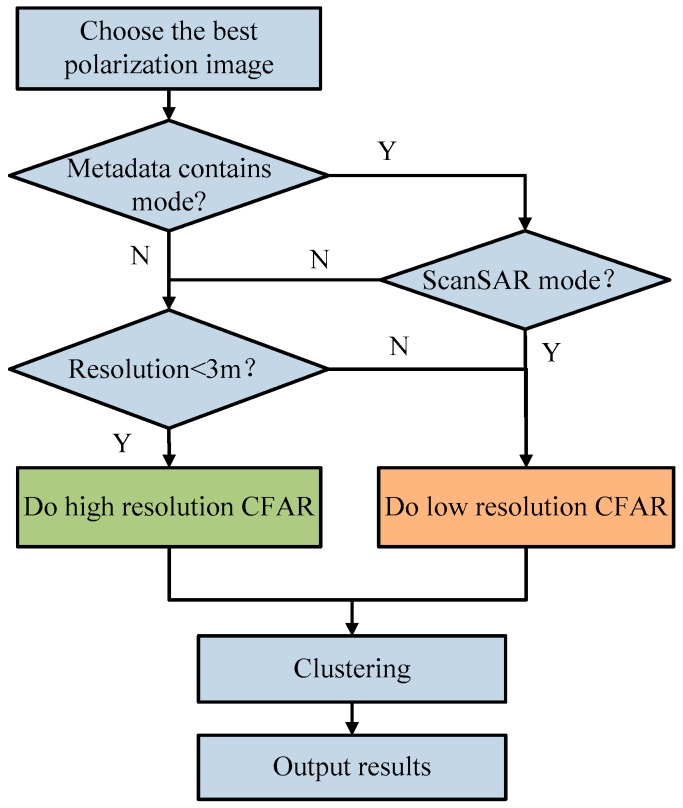
Detailed flow of ship candidate detection. Y (yes), N (no).

After land masking, land areas are removed from SAR imagery. Thus, prescreening algorithms will focus on the ocean easily. In designing and implementing prescreening, care needs to be taken with the type of SAR imagery. Prescreening algorithms designed for one type of imagery may not be appropriate or need to be adjusted for a different type of imagery [2,13]. In this respect, spatial resolution is obviously a critical factor as it is the core objective of SAR sensors. Other factors, such as the imaging mode, incidence angle or polarization channel of SAR imagery are also important. In this paper, taking into account the imaging mode, incidence angle, polarization channel of spaceborne SAR imagery and ship size, we implement the ship candidate detection by applying different detection strategies to different resolution imagery. Figure 3 shows the detailed flowchart of ship candidate detection proposed in this paper. The prescreening stage can be concluded into four steps basically.

#### 2.2.1. Acquiring the Metadata of SAR Imagery and Specifying Ship Size

In this step, the metadata, including the SAR imaging mode, spatial resolution, incidence angle, pixel size, and available polarization channels, are extracted from the SAR data. Parameters of ship candidate detection are based on these metadata.

As the core objective of SAR sensors, the spatial resolution will determine which detection algorithm is applied. Since the current advanced SAR sensors (TerraSAR-X, COSMO SkyMed, etc.) usually provide a resolution higher than 3 m while early SAR sensors cannot, 3 m is considered as the threshold to distinguish high resolution and low resolution imagery in this paper. Two distinct CFAR strategies are applied to high resolution and low resolution SAR imagery respectively. The imaging mode also helps to determine the CFAR strategy.

This paper focuses on the single polarization channel imagery. However, if more than one polarization channel is present, different techniques may be applied. Both the incidence angle and available polarization channels are used to choose the best polarization channel image for ship detection. At low incidence angles ships are easier to detect in the cross-polarized (HV/VH) since the clutter levels are lower than co-polarized (HH/VV) while the ship radar cross section (RCS) remains the same [15]. Thus, the cross-polarized data is used for low incidence angles, and co-polarized data is used for high incidence angles. The threshold value to distinguish high or low incidence angles is 45° [2]. In the case where only co-polarized channels are available (HH + VV), HH is always preferred because of its lower clutter levels [13,15]. The decision to use the cross-polarized or co-polarized channel is based on per tile, which will be discussed in the next step.

In the land masking stage, both pixel size and ship size will determine the downsampling rate. They also determine the sizes of CFAR windows (background, guard, and target). If ship size is unknown, one can use the empirical value. In general, the maximum length of ships is 300 m, and the maximum width is 60 m. The pixel size is essential to calculate the distance in SAR imagery. It is also important for locating the azimuth ambiguities in the ship discrimination.

#### 2.2.2. Breaking up the Image into Tiles of Size 512 × 512

In this step, the image will be broken up into tiles of size 512 × 512. Swath widths of large footprint images, such as ScanSAR mode images, can reach hundreds of kilometers. Thus, their incidence angles vary considerably between near and far range. For example, the swath width of RADARSAT-2 wide ScanSAR mode imagery is 500 km × 500 km, and the incidence angle varies from 20° to 49°. In general, RCS of the ocean decreases with increasing incidence angle. Thus, the whole image is a nonuniform image, which aggravates the ship detection. Breaking up the image into tiles contributes to alleviating this aggravation. It allows for the efficient processing of very large data by reducing memory consumption and optimization program design.

#### 2.2.3. Applying Different CFAR Algorithms to Each Tile Based on the Metadata

In this step, we apply different ship detection strategies to each tile based on the metadata. The ship detection algorithms are based on the CFAR detector. An important step of CFAR is to design a distribution model for the background (between guard window and background window), i.e., to specify an associated probability density function $f_{pdf}\left(x\right)$ [2,25,32]. Once $f_{pdf}\left(x\right)$ has been specified, the probability of false alarm (PFA) for the threshold T is given by

(2) $$PFA=1-\int_{-\infty }^{T}f_{pdf}\left(x\right)dx=\int_{T}^{\infty }f_{pdf}\left(x\right)dx.$$

In the case where the spatial resolution is inferior to 3 m, we apply low resolution CFAR to SAR imagery. The core of low resolution CFAR is a CFAR detector based on K-distribution. K-distribution is widely used because of its well-known modeling ability [2,33]. The integral of the K-distribution is solved numerically. The threshold is determined by a binary search in this paper. The target window is set to be a similar size of the maximum length of ship targets, the guard window is set to be a double size of the target window, excluding the ship from the background window, and the background window is set to be 10% larger than the guard window. Assuming that the maximum length of ships is 300 m, then the size of the target window is 300 m, the size of the guard window is 600 m, and the size of the background window is 660 m. The window sizes are then calculated in terms of pixels by normalizing sizes using the pixel size. Different from many previous CFAR algorithms, the size of the target window is greater than one pixel. The CFAR windows slide with a step of the target window rather than one pixel. Each pixel in the target window is compared with the threshold calculated by the same background. If a pixel value is higher than the threshold, it is determined to be a ship pixel. Pixels in a same target window only need to calculate a threshold once. This speeds up the algorithm considerably and has little influence on the detection of median or large size ships. The performance in the detection of small targets depends on the ship detectability and the accuracy of the clutter statistical model.

**Figure 4:**
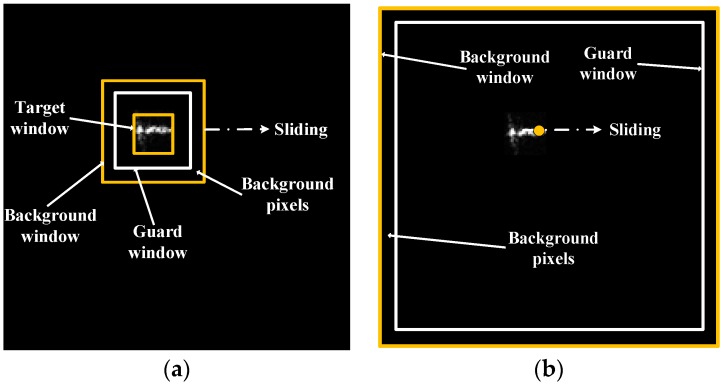
Comparison of sliding windows of low resolution CFAR (Constant False Alarm Rate) (**a**) and high resolution CFAR (**b**), the black block represents an image tile.

In the case where the spatial resolution is under 3 m, we apply high resolution CFAR to SAR imagery. Similarly, the core of high resolution CFAR is a CFAR detector based on K-distribution. However, unlike low resolution CFAR, the background pixels of high resolution CFAR are fixed to come from the border ring of the tile. One tile corresponds to a same background. Actually, the background window is the outer edge of the tile (512 × 512), the guard window is about 10% smaller than the background window (450 × 450), and the target window is 1 pixel. The target window (1 pixel) slides in the whole window (including the background). Thus, the ship will be detected if it happens to be in the background area. The reason why we can do this is that the imaging area is quite small in one tile for high resolution SAR imagery (pixel size under 1 m usually). One tile often contains no more than one ship target. Thus, the background clutter is roughly stationary throughout the tile. This improves the speed efficiently and will not degrade the detection performance. Figure 4 shows the difference between sliding windows of low resolution CFAR and high resolution CFAR. The black block represents an image tile in Figure 4.

It should be noted that the proposed method does not check whether the background window is free from ships in both the high and low resolution cases. Thus, ship targets may be missed if there are ship targets located in the background area. A solution should be developed for this problem in the future. Besides, there are many efficient ship detection methods using multi-polarization data [3,34,35,36]. Though this paper focuses on the single polarization channel imagery, it will exploit more polarization channels in the future.

#### 2.2.4. Clustering and Outputting the Results

After CFAR detection, detected pixels are not clustered. We employ an erosion algorithm to remove isolated pixels firstly and then group the detected pixels into a single detection by a dilation method. After the detected pixels are grouped together, the median point of each target is calculated in the binary image. Then, each candidate chip which centers at the median point is extracted from the original image. The size of the chip is set to be as twice as the maximum length of ships. This ensures that the whole ship target is contained in the chip. Each detection result is presented as an image chip of a same size finally.

## 3. Ship Discrimination

Since ship candidate detection is a “fast but dirty” stage [13], the preliminary detection result is difficult to avoid false alarms. Most ship detection systems have a discrimination stage following the prescreening stage. The idea of the discrimination stage is to reduce the false alarm rate. Discrimination algorithms usually operate on an image chip which contains a ship candidate and its local surroundings. Generally, discrimination methods can be divided into methods based on features and knowledge.

**Figure 5:**
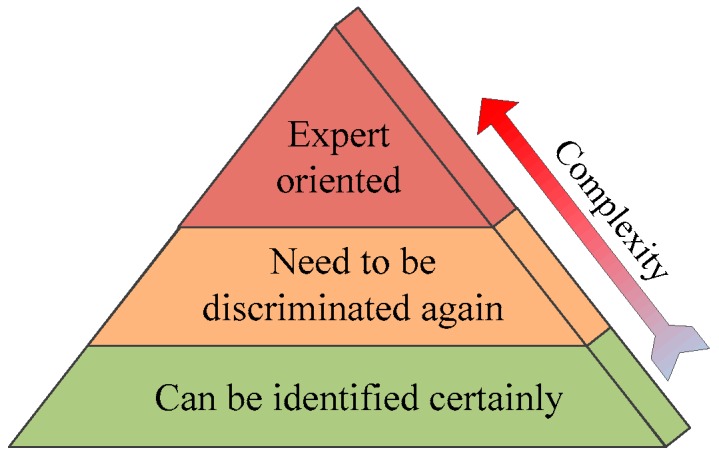
Basic idea underlying the comprehensive ship discrimination.

As most ship targets and false alarms present different characteristics, discrimination methods based on features play a most important role and perform well in ship discrimination. However, discrimination methods based on features cannot identity false alarms with same features like ship targets, e.g., azimuth ambiguities. Besides, automation discrimination cannot guarantee the correctness of discrimination results. Thus, a better way for ship discrimination is to take into account various discrimination methods. In this paper, we propose a comprehensive ship discrimination method. The basic idea underlying the proposed method is based on discrimination complexity as shown in Figure 5. Above all, it rejects false alarms which can be identified certainly by certain discrimination based on features (CeD). Then, confidence discrimination based on features (CoD) is applied. Each discrimination result will be marked with a confidence probability, followed by manual eye inspection. Finally, it proposes to discriminate false alarms caused by azimuth ambiguities, which need expert knowledge.

### 3.1. Discrimination Based on Features

Current features can be generally divided into four categories: textural features, size features, contrast features and polarimetric features [37]. Among these features, Lincoln Laboratory Discrimination Features and ERIM (Environmental Research Institure of Michigan) Discrimination Features are the most widely used [38,39]. In this paper, size features are considered to be more accurate to identify false alarms. Generally, size features can reject certain false alarms accurately. Thus we categorize discrimination based on features into certain discrimination and confidence discrimination based on features. The former refers to discrimination methods whose results are correct. In particular, it means that rejected targets are false alarms absolutely. The latter means that discrimination results may not be totally correct. Certain discrimination is applied to exclude certain false alarms firstly. Then confidence discrimination is applied and the confidence probability of each discrimination result is calculated.

#### 3.1.1. Certain Discrimination Based on Features

Features used for CeD are usually size or shape features. Textural or contrast features may vary considerably in SAR images. Different from these features, size or shape features are more reliable in SAR images. They can be used to reject some false alarms certainly. Among them, length L, width W and aspect ratio AR of ships are considered to be most intuitive. Thus, these three features are used for CeD. Usually, size and shape features are difficult to extract for ships that have problems with sidelobes. In this paper, a refined ship segmentation method [40] developed by our research group is used to alleviate sidelobes’ effect. It is original from Radon transformation [41] and improved 2D Otsu method [42]. Then L and W are extracted from the segmentation result. Aspect ratio AR is calculated by L and W.

Empirically, the length of ship targets is no longer than 300 m in most cases (Certain ships can be longer than 300 m, e.g., aircraft carriers, and this threshold can be adjusted according to users’ need), and the width is no longer than 60 m. However, both the measured length and width are usually larger than the real value because of sidelobes. Smearing or defocusing caused by the movement of ships also enlarges the length or width. Thus, the upper limit of L or W are set larger, 360 m and 80 m, respectively. If L or W is beyond the range, it may be not a ship. At the same time, because of designed requirements, the aspect ratio of ships is less than 10. As L and W are enlarged, the aspect ratio of ship targets in SAR images is usually smaller. We set the threshold of aspect ratio as 9 in this paper. If aspect ratio is larger than 9, the target is not considered to be a ship.

#### 3.1.2. Confidence Discrimination Based on Features

During CeD, some ship candidates are discarded as they are considered to be false alarms with high degree of certainty. However, the remaining detection results can still contain false alarms. Supervised learning methods such as QD (Quadratic Distance), SVM (Support Vector Machine), BNN (Bayesian Neural Network) and QPD (Quadratic Polynomial Distance) [43,44,45] usually include two steps: training and testing. However, they are not easy for ship discrimination because training and testing data can be collected under different background, by different sensors, in different weather conditions. The training requires enough representative data. However, it is not a tractable work usually.

After visualizing the samples collected from SAR images, it is found that ship targets will group together and clutters will scatter and far away from targets in the feature space. It is more suitable to use a clustering method as K-means to divide the candidates into target and clutter groups. Thus, we can use the rules inside the candidates instead of learning it from other candidates to distinguish between target and clutter. In this paper, the K-means discrimination method proposed in [46] is modified to apply confidence discrimination.

In this paper, the three discrimination features used are standard deviation, peak power and average power of target areas.

The standard deviation σ is defined as follows:

(3) $$\begin{array}{l} \sigma =\sqrt{\left(S_{2}-{S_{1}}^{2}/N\right)/\left(N-1\right)}\\ S_{1}=\sum I\left(x,y\right)\;,\;\left(x,y\right)\in \text{Chip}\\ S_{2}={\sum \left[I\left(x,y\right)\right]}^{2},\;\left(x,y\right)\in \text{Chip} \end{array},$$

where *N* is the number of pixels in the chip, “chip” means the whole chip. Usually, there are large fluctuations in a chip containing ship targets. As a result, the standard deviation of a chip containing ship targets is much larger than clutter chips. The use of single size chips can bias the standard deviation. Candidate ships with different dimensions will have different standard deviations, all other things (including chip size) being equal. This bias has been calibrated in this paper.

The average power of target areas Apt is defined as follows:

(4) Apt=(1/N)∑I(x,y),   (x,y)∈Target blob,

where *N* is the number of pixels in the target blob, target blob is the result after an AND operation of the original chip and the binary chip achieved by the refined ship segmentation method [40]. This feature is discriminative due to that it is uncorrelated to size of targets. For target chip, *Apt* is much larger than that of clutter chips.

The peak power of target areas $I_{\text{max}}$ is defined as follows:

(5) $$I_{\text{max}}=\max\left(I\left(x,y\right)\right),\;\left(x,y\right)\in \text{Target blob},$$

Its reason to be discriminate is similar to Apt.

To use K-means discrimination method, features should be normalized into interval [0, 1] firstly to eliminate difference between dimensions. Each feature is divided by the maximum without any transformation. Ideally, target group will gather around [1, 1, 1] while clutters group around [0, 0, 0] in the feature space after normalization. The number of clusters is set to be 2. Their initial centers are predefined as [1, 1, 1] and [0, 0, 0], respectively. Thus, the target points will be the first cluster while clutter points will be the second one as we hope.

A problem for K-means discrimination method is that results are not always that correct because of variety of clutters and ship targets. It is distinguished to the certain discrimination method. Besides, wrong discrimination results are difficult to be identified. We do not know which discrimination result is more reliable. Further discrimination based on the results is difficult to start with. Thus, it is essential to design a strategy to estimate the confidence level of each discrimination result in which the most unreliable results are identified.

To cure the above problems, we propose a confidence method to identify wrong discrimination results. After K-means clustering, we calculate the difference $d_{i}$ between distances from each feature point $t_{i}$ to the 2 final cluster centers $u_{1}$ and $u_{2}$, $d_{i}=\left\|t_{i}-u_{1}\right\|-\left\|t_{i}-u_{2}\right\|$. Obviously, the maximum value of $d_{i}$ is the diagonal length D of the feature space. If the dimension is n and features are normalized into interval [0, 1], then $D=\sqrt{n}$. The confidence probability is defined as follows in this paper,

(6) $$P_{i}=\text{ ​}\left|d_{i}\right|/D=\left|\left\|t_{i}-u_{1}\right\|-\left\|t_{i}-u_{2}\right\|\right|/\sqrt{n}.$$

With $P_{i}$ increasing, $\text{​}d_{i}$ is larger, which means that the discrimination result is more reliable; on the contrary, $\text{​}d_{i}$ is smaller which means that tiny noise can lead to different discrimination results and the discrimination result can be unstable and unreliable. We set a threshold $P_{0}$. If $P_{i}<P_{0}$, the discrimination result is considered to be doubtful and need to be discriminated again,

(7) $$P_{i}=\text{ ​}\left|d_{i}\right|/D=\left|\left\|t_{i}-u_{1}\right\|-\left\|t_{i}-u_{2}\right\|\right|/\sqrt{n}<P_{0},$$

that is to say, any target which matches $d_{i}=\left\|t_{i}-u_{1}\right\|-\left\|t_{i}-u_{2}\right\|\in \left(-P_{0}\sqrt{n},P_{0}\sqrt{n}\right)$ need to be discriminated again; otherwise, the i th discrimination result is considered to be correct. Actually, the decision plane is a hyperboloid whose focus are final cluster centers $u_{1}$ and $u_{2}$, distance difference is $P_{0}\sqrt{n}$. Obviously, the CeD method proposed in this paper is able to provide a quantitative estimation for the confidence level of each result. Doubtful results can be identified easily. Usually, they will be checked by eye inspection as its number is small. The results from eye inspection are considered to be correct and their confidence probabilities are 1.

Another problem for K-means discrimination method is that if only one class of candidates is present (i.e., only real ships or only false alarms), it should not be applied. Otherwise, some real ships may be incorrectly classified as false alarms (or vice versa) by the K-means algorithm. Considering that if the number of candidate chips is small, the probability of only one class is high. Otherwise, the probability of only one class is low. Thus, only when the number of candidate chips is large enough, CoD is applied. Otherwise, CoD will be skipped and all candidate chips will be checked by eye inspection.

### 3.2. Removal of Azimuth Ambiguities

**Figure 6:**
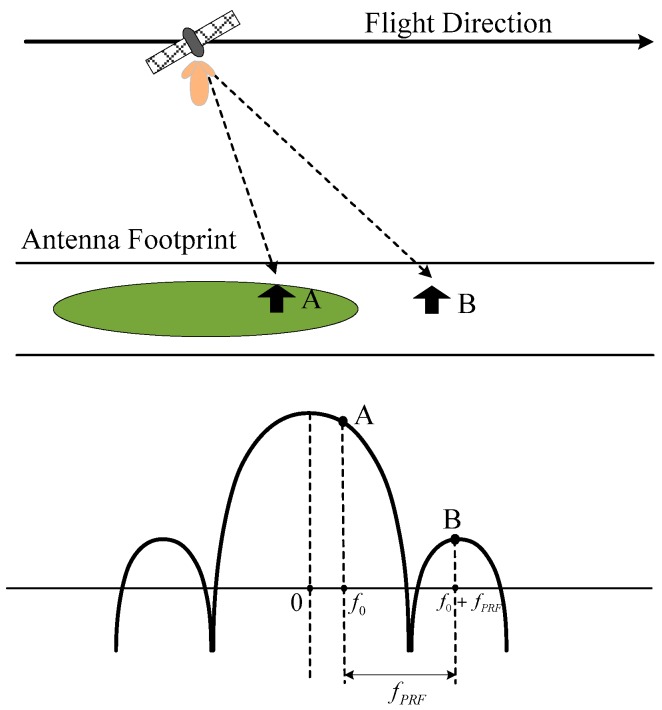
Illustration of azimuth formation in synthetic aperture radar (SAR) images (Points A and B have equal Doppler histories due to aliasing) [50].

**Figure 7:**
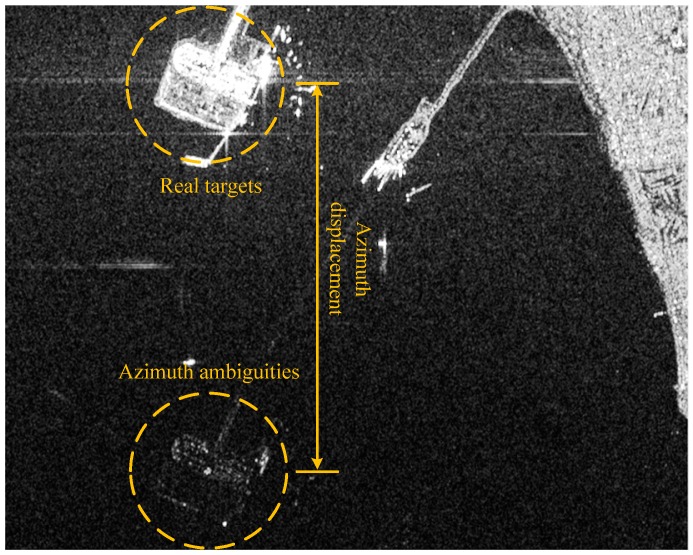
Example of azimuth ambiguities in SAR imagery (Roberts Bank Port at Georgia Strait, Vancouver, acquired by RADARSAT-2 in Standard mode).

Experience shows that azimuth ambiguities are today a real problem for ship detection [47,48]. Generally, a too high pulse repetition frequency (PRF) may produce the overlap of two successive returns causing range ambiguities. However, SAR systems are usually designed to avoid or reduce range ambiguities [49]. On the other hand, a too low PRF may cause the effect that Doppler frequencies higher than the PRF are folded into the azimuth spectrum causing aliasing [50]. As shown in Figure 6, points A and B have equal Doppler histories due to aliasing. If a target in B is much stronger than whatever in A, and if the target in B is not cancelled out by the antenna pattern during point A’s illumination time, then it is possible that a ghost image of the target in B will be visible in point A. This ghost is called azimuth ambiguity. If range ambiguities occur, they usually are far away from the source, on the order of 100 km [49]. Thus, they are difficult to recognize and have less influence on ship detection. However, typical azimuth ambiguity distances are 5~10 km [49]. This will have a significant impact on ship detection. Areas near the coast can be strongly affected by land-based sources, as depicted in Figure 7. In cases where the clutter level is lower mentioned in Section 2, azimuth ambiguities can occur more easily. If unrecognized, azimuth ambiguities will give rise to false alarms.

Azimuth ambiguities in SAR images are spatially displaced in azimuth directions at approximate locations [49,50],

(8) $$\Delta D_{AZ}\;=\;n\frac{\lambda R_{s}}{2V}f_{PRF},$$

where $\Delta D_{AZ}$ is the azimuth displacement, λ is the wavelength, V is the satellite velocity, $R_{s}$ is the slant range, and $f_{PRF}$ is the PRF.

Azimuth ambiguities are difficult to be recognized by discrimination methods based on features because of their special origins. Usually, they are similar to their source targets in some features. A popular way to identify azimuth ambiguities is to check whether there is a stronger source target present at the azimuth displacement. If either side has a detect of stronger power than the source target, it is considered to be an ambiguity and discarded. They are compared manually. In general, the candidate with the strongest power is regarded as the ship target. Both first and higher order ambiguities will be removed.

**Figure 8:**
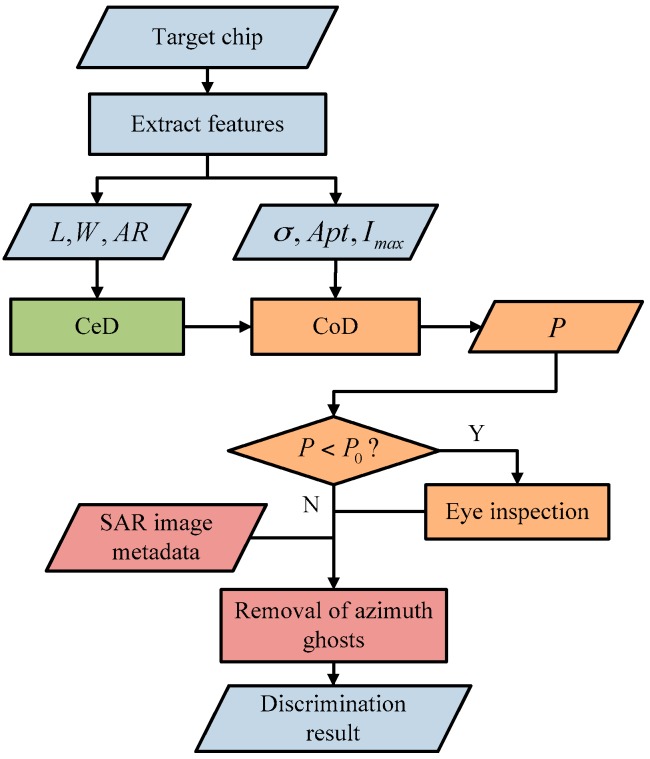
Detailed flow of ship discrimination. L (length), W (width), AR (aspect ratio), σ (standard deviation), Apt (average power of target areas), $I_{\text{max}}$ (peak power of target areas), CeD (certain discrimination based on features), CoD (confidence discrimination based on features), P (confidence probability), $P_{0}$ (threshold of P), Y (yes), N (no).

In summary, the detailed flowchart of ship discrimination proposed in this paper is shown as Figure 8.

## 4. Experimental Results and Discussion

In this section, the experimental results obtained using the proposed scheme are introduced and discussed. Several images will be used to test our methods. All experiments were carried out by using a PC with i3 Dual-Core CPU of 3.3 GHz and memory of 4 GB. The codes are written without optimization.

### 4.1. Ship Candidate Detection

#### 4.1.1. Land Masking

**Figure 9:**
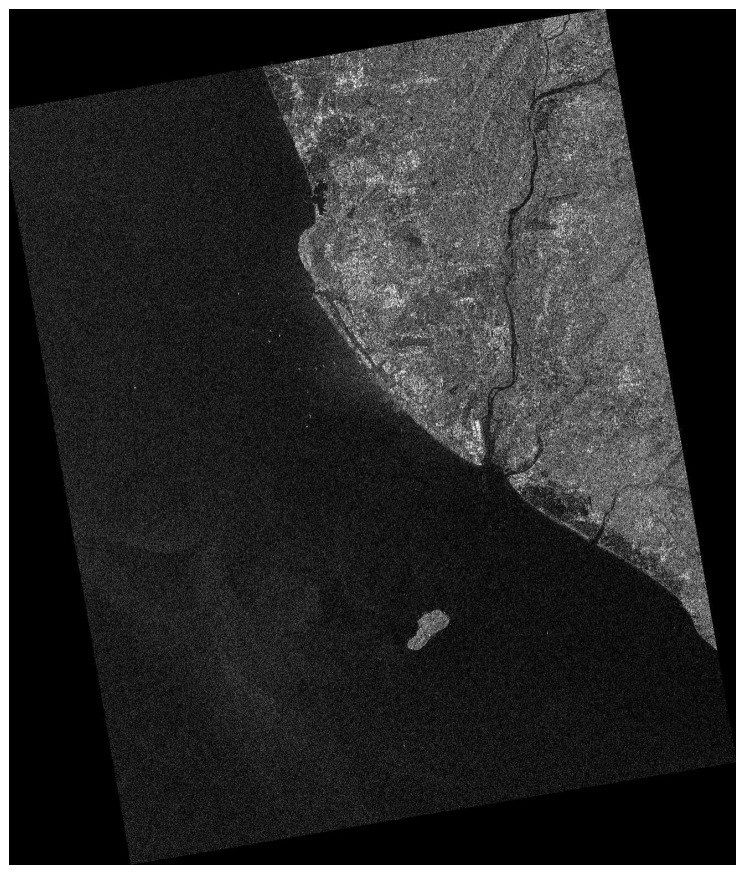
RADARSAT-1 image (Kaohsiung, Taiwan, acquired by RADARSAT-1 in FINE mode).

Figure 9 shows the RADARSAT-1 image whose imaging area is Kaohsiung, Taiwan. Its imaging mode is FINE, polarization channel is HH, size is 8758 × 10,472, spatial resolution is 8 m, and pixel size is 6.25 m. The incidence angle varies from 37° to 48°. Here only the HH channel image is provided. Its clutter level is low and azimuth ambiguities can occur easily. The island and rivers in this image can influence land masking. The downsampling rate R=shipwidth/pixelsize=9.6.

**Figure 10:**
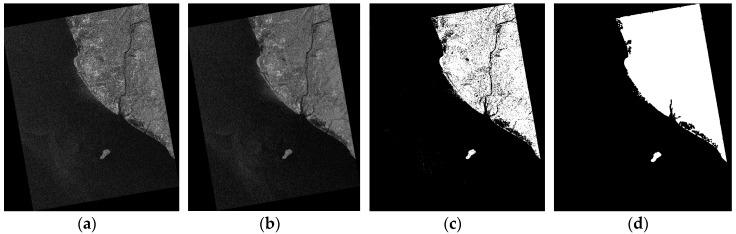
Land masking results of RADARSAT-1 image: (**a**) The image after downsampling; (**b**) The image after median filtering; (**c**) The image after thresholding; and (**d**) The land mask.

Experimental results of the RADARSAT-1 image are shown in Figure 10. The island is wiped out successfully and no ship targets are taken as land areas. The experiment takes only seconds. From the results, a preliminary conclusion can be made that the algorithm proposed in this paper is fast and efficient. The image after land masking is suitable for ship detection.

**Figure 11:**
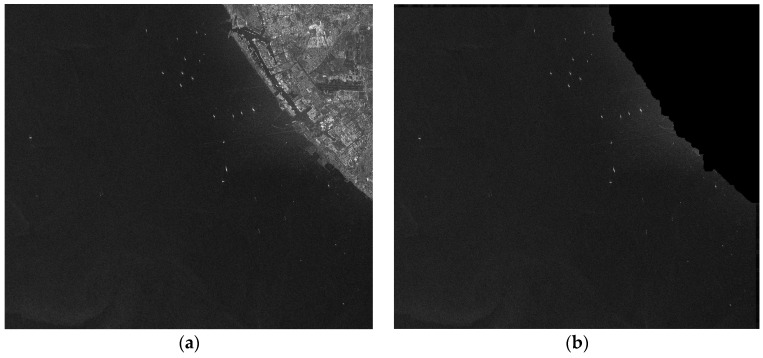
Land masking results of RADARSAT-1 ROI, (**a**) The image after cutting; and (**b**) The image after land removed.

We intercept a region of interest (ROI) containing most of ships from the RADARSAT-1 image, as shown in Figure 11a. The corresponding image whose land areas are removed is shown in Figure 11b. It can be see that land areas are removed appropriately and ship targets are kept well. Figure 11b will be used in the following step.

#### 4.1.2. Prescreening

**Figure 12:**
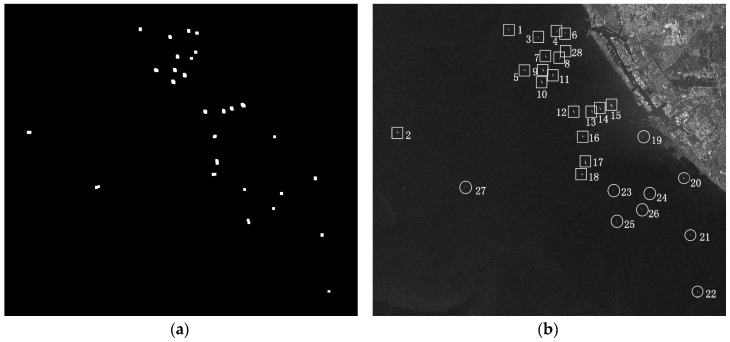
Prescreening results of RADARSAT-1 ROI, (**a**) The result after clustering; (**b**) The result marked in the ROI.

ROI of RADARSAT-1 is tested in this section. Its size is 3964 × 3500. There are 19 ships in total according to eye inspection. As its resolution is 8 m, inferior to 3 m, the low resolution CFAR method is applied. Thus, the size of target window is 300 m × 300 m, or 48 × 48 in pixels; the size of guard window is 600 m × 600 m, or 96 × 96 in pixels; the size of background window is 660 m × 660 m, or 106 × 106 in pixels. *PFA* is set as 1 × 10^−5^. Detection results are shown in Figure 12. Figure 12a is the result after clustering. The result contains 28 candidates and 19 of them are real ships by visual inspection. In Figure 12b, false alarms are marked with a circle and real ships are marked with a rectangle. It costs 2.7 s.

Similar experiments are conducted on other SAR image data, produced by TerraSAR-X, RADARSAT-2 (ScanSAR(Wide)), RADARSAT-2 (Fine), RS-1 and RS-3 of China. They are all listed in Table 2. Their parameters, including imaging mode, resolution, polarization mode, image size and pixel size, are also present in Table 2. It should be noted that RADARSAT-2 data contains both HH and HV mode images. However, both their incidence angles are considered to be low because most of the sea area in the Radarsat-2 images is under 45°. As mentioned in Section 2, it is easier to use the cross-polarized mode images for ship detection. Thus, the HV mode is used in these experiments. Based on these parameters, low or high resolution CFAR method and its window sizes are determined. *PFA* is set as 1 × 10^−5^ (It should be higher enough to detect all potential targets). Concrete parameters for each experiment are presented in Table 3.

Experimental results are listed in Table 4, including time consumption, number of targets detected, real ship targets (the ground truth was obtained by visual inspection), correct targets detected, false alarms, targets missed and FoM (Figure of Merit). FoM is defined as

(9) $$FoM=\frac{correct\;targets\;detected}{real\;ship\;targets+false\;alarms}.$$

Larger FoM represents better performance. From Table 4, we can see that no ship targets are missed in the six experiments using our method. The adaptive ship candidate detection is able to detect all ship targets. However, there are false alarms when sea condition is complex. Compared to some other high-precise ship detection methods, the false alarm rate can be higher by our method.

**Figure 13:**
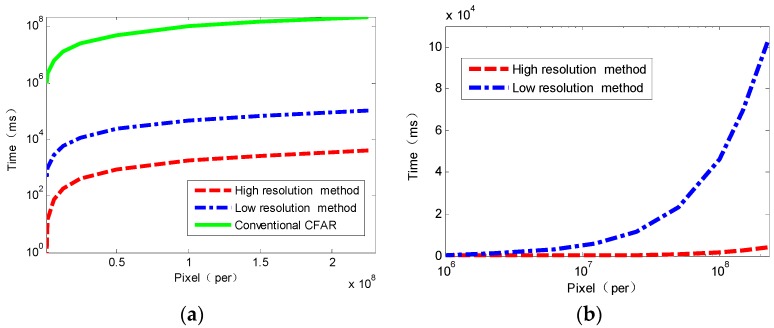
Comparison of time consumption between the proposed method and conventional CFAR (**a**), the high and low resolution method (**b**).

Time consumption of our methods is on the order of seconds. Compared with conventional CFAR methods (K-distribution based, CFAR windows slide with a step of one pixel) whose work lasts tens of minutes or more than one hour, the proposed method can finish in a real time fashion when applied to large size images. Conventional CFAR here means that we apply the same CFAR (same distribution mainly) over each pixel of the image without using our method, particularly its CFAR window setting is different. The biggest time-saver is the frame proposed in Section 2.2. In order to evaluate the time consumption in a more detailed manner, we derive the results of experiments on images whose size ranges from 1000 × 1000 to 15,000 × 15,000. Figure 13 presents the comparison of time consumption between the proposed method and conventional CFAR. It is obvious that the proposed method (High or low resolution CFAR) is much more economic than the conventional CFAR method in time consumption. For an image of 200 million pixels (a popular size of spaceborne SAR image), conventional CFAR costs about 60 h, low resolution method fewer than 2 min and high resolution method about 4 s. Thus, the proposed method can be completed in seconds for most SAR imagery.

In summary, ship candidate detection method proposed in this paper is able to detect ship targets in a fast, efficient and robust way. However, false alarms may occur.

### 4.2. Ship Discrimination

In this section, preliminary detection results containing false alarms in prescreening stage (Test image ID 1 and 6) are used to validate our discrimination method. Ship candidate detection and ship discrimination methods proposed in this paper are expected to be a complete process as we hope. Each prescreening result is presented as a chip in ship discrimination with a size of 100 × 100 (in pixels).

#### 4.2.1. CeD

Length L, width W and aspect ratio AR are extracted from the 28 candidates of test image ID 1, as shown in Table 5. L and W are measured in pixels. It can be seen that L of each target is smaller than 360 m, W smaller than 80 m, and aspect ratio AR smaller than 9. There is no exception for all targets. However, it does not mean there is no false alarms any more. All targets will be analyzed next.

Similar experiments and analysis are conducted on the detection results of test image ID 6, and it is found that no exception occurs. All targets are considered to be ship targets.

#### 4.2.2. CoD

**Figure 14:**
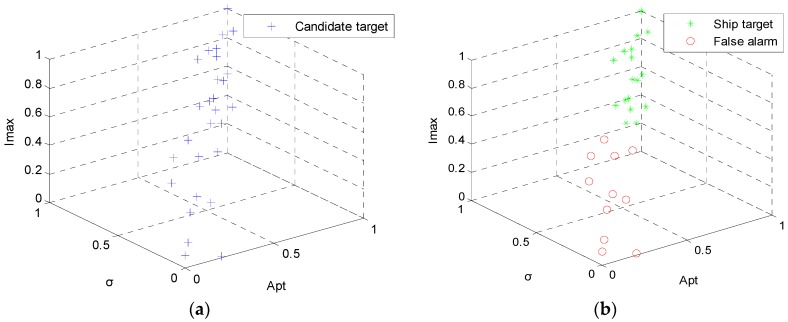
Candidate target feature space (**a**) and preliminary discrimination results (**b**) for test image ID 1.

In this section, CoD method is applied to the 28 candidates. The three features are normalized into interval [0, 1] firstly. Figure 14a shows the feature space. Each blue + represents a candidate target. These candidate targets are clustered into 2 clusters by K-means discrimination method. The preliminary discrimination results are shown in Figure 14b. Green * near [1,1,1] represents ship target and red ○ near [0,0,0] false alarm.

**Figure 15:**
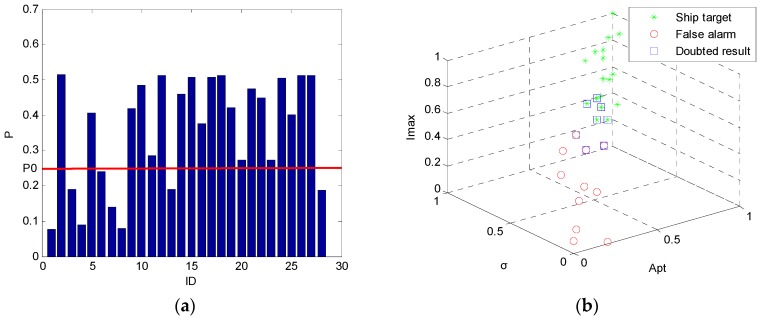
Confidence probabilities (**a**) and doubted results (**b**) for test image ID 1.

As shown in Table 6, CoD method returns 11 false alarms and 17 ship targets in total. √ represents ship target and × represents false alarms. We calculate the confidence probability of each discrimination result via the Formula (6). Figure 15a provides a bar graph to illustrate confidence probability more briefly. We set the threshold $P_{0}$ = 0.25 in this paper (It can be adjusted according to users’ need). It is clear that there are 20 discrimination results whose confidence probabilities are higher than $P_{0}$. They are considered to be correct. On the other hand, there are 8 discrimination results whose confidence probabilities are lower than $P_{0}$. They are considered to be doubtful and need to be discriminated further. These 8 results are 1st, 3rd, 4th, 6th, 7th, 8th, 13th, and 28th. They are marked with blue □ in Figure 15b. As shown in Figure 15b, they are so close to each other that they can get wrong.

Eye inspection is applied to these 8 candidate targets. It is found that the 4th, 6th, and 28th candidate targets are taken to be false alarms while they are actually ship targets. The previous decisions on 1st, 3rd, 7th, 8th, and 13th candidate targets are correct.

A final conclusion is present in Table 7, and each confidence probability is listed. From the table, there are 20 ship targets and 8 false alarms according to the discrimination method based on features.

**Figure 16:**
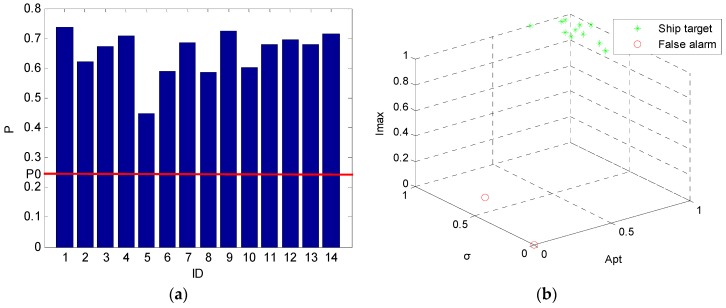
Confidence probabilities (**a**) and discrimination results (**b**) for test image ID 6.

Similar operation can be applied to prescreening results of test image ID 6. The discrimination results and confidence probabilities are shown in Figure 16. There are 12 ship targets and 2 false alarms in total. The 2 false alarms are the same candidates in test image ID 6 in Table 4. Their confidence probabilities are all above 0.25. Thus, they are all reliable.

#### 4.2.3. Removal of Azimuth Ambiguities

Azimuth ambiguity is an exception for discrimination method based on features as it has same features of source targets. Thus, the discrimination results based on CeD and CoD can be polluted by azimuth ambiguities.

Knowing that under the FINE imaging mode for the RADARSAT-1, the wavelength λ = 0.05657 m, satellite velocity V = 7062 m/s, PRF $f_{PRF}$ = 1256.98 Hz, slant range $R_{s}\approx h/\cos\eta$, h = 793,000 m which is the satellite height, η = 37° which is the incidence angle at the near range, according to the Formula (8), azimuth displacement (*n* = 1),

$$\Delta D_{AZ}\;=\;n\frac{\lambda R_{s}}{2V}f_{PRF}\;\approx \;4900\;m.$$

**Figure 17:**
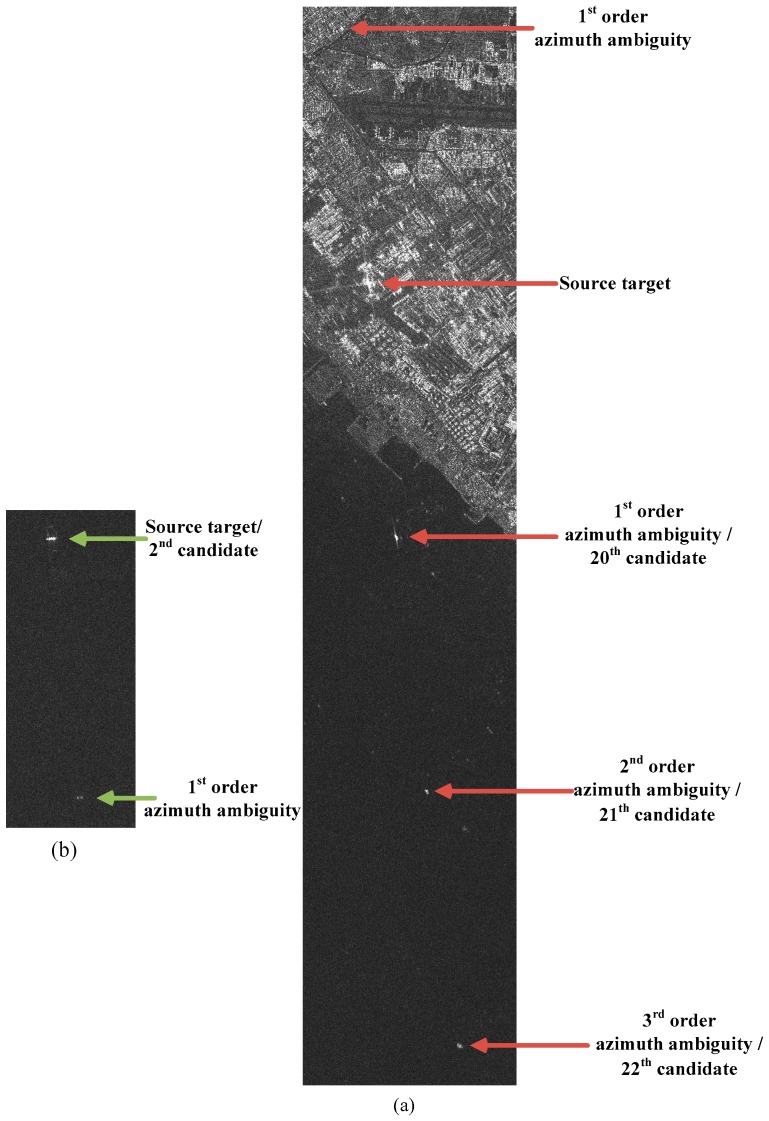
Azimuth ambiguities caused by a ship target (**a**) and land-based sources (**b**) in test image ID 1.

As pixel size is 6.25 m, the displacement in the image can be calculated as 4900/6.25=784. That is to say, candidate targets which are 784 pixels apart in the azimuth direction can be azimuth ambiguities. It should be noted that the value of 4900 m is only valid at incidence angle 37°. We checked the candidates, and found that both sides of the 20th target have a similar target and the target on the land is the strongest one, as shown in Figure 17a. Thus, the target on the land is considered to be the source target. The 20th target is a false alarm, though it is considered to be a ship target by CeD and CoD. The final detection results are outputed and matched to the ground truth. The false alarms refer to the same candidates in image ID 1 in Table 4.

Attention should be paid to that the source target on the land generates the 2nd azimuth ambiguity (21st candidate target) and 3rd azimuth ambiguity (22nd candidate target) as shown in Figure 17a. The 2nd candidate target also forms 1st order azimuth ambiguity. However, they are all cascaded by CFAR or CoD as their low contrast. The 20th candidate target, however, is very similar to a real ship target either in contrast features or shape and size features. Discrimination methods based on features cannot distinguish it any more. It also confuses human eyes if the prior information is not given. Thus, the removal of azimuth ambiguities is an essential problem that should be considered.

## 5. Conclusions

In this paper, we present a practical adaptive ship detection scheme for spaceborne SAR imagery. It is able to process a wide range of spaceborne SAR data. The aim of this scheme is to solve the practical problems in ship detection. We have reviewed the general flow of ship detection in spaceborne SAR imagery and its problems. Accordingly, two main works are completed in this paper as follows.

- The first one is an adaptive ship candidate detection method. It has two advantages mainly. The first concerns that the method is able to process several kinds of SAR images collected by different sensors. The second is related to that the method is able to detect potential ship candidates in a fast, efficient and robust way. Both make it a practical method for ship candidate detection in spaceborne SAR imagery.
- The second one is a comprehensive ship discrimination method. It also has two advantages mainly. The first is that it is designed to discriminate various possible false alarms in the ship candidates based on confidence level and complexity. It can integrate various discrimination methods (eye inspection included) efficiently. The second is that it can provide a confidence probability for each discrimination result instead of a simple True/False decision.

Thus, a scheme of this kind may become viable for adaptive ship detection in spaceborne SAR imagery. However, as there is a lack of validation data (e.g., AIS), some further validation of the proposed scheme needs to be done in the future. Contents presented in this paper are preliminary results. A new ship detection system wihch will provide user interface with data and results is currently under development partly based on this scheme.

## Figures and Tables

**Table 1:** List of current typical spaceborne synthetic aperture radar (SAR) systems.

Sensor	Band	Polarization	Resolution	Swath	Incidence Angle	Country
RADARSAT-2 [13]	C	Quad	3~50 m	10~500 km	10°~59°	Canada
TerraSAR-X [6]/TanDEM-X [20]	X	Quad	1~50 m	10~500 km	15°~60°	Germany
COSMO SkyMed 1–4 [21]	X	Quad	1~100 m	10~200 km	35.5°~40.5°	Italy
Sentinel-1a/1b [14,22]	C	Dual	5~40 m	20~400 km	19°~47°	EU
RISAT [23]	C	Quad	2~50 m	10~240 km	10°~54°	India
ALOS PALSAR-2 [3]	L	Quad	1~100 m	25~490 km	8°~70°	Japan
Kompsat-5 [24]	X	Dual	1~20 m	5~100 km	20°~45°	Korea

**Table 2:** Experimental data and their parameters.

Image ID	Sensor Type	Imaging Mode	Resolution	Polarization	Image Size	Pixel Size
1	RADARSAT-1	Fine	8 m	HH	3964 × 3500	6.25 m
2	TerraSAR-X	Stripmap	3 m	HH	10,859 × 6950	-
3	RADARSAT-2	ScanSAR(Wide)	100 m	HH/HV	660 × 360	50 m
4	RADARSAT-2	Fine	8 m	HH/HV	1437 × 1046	6.25 m
5	RS-1	-	5 m	-	2752 × 2393	2.5 m
6	RS-3	-	≤3 m	-	4559 × 4559	-

**Table 3:** Parameters for each experiment. *PFA* is the probability of false alarm.

Image ID	CFAR Method Used (High or Low Resolution)	Background Window Width (in Pixels)	Guard Window Width (in Pixels)	Target Window Width (in Pixels)	*PFA*
1	Low	106	96	48	1 × 10^−5^
2	High	512	450	1	1 × 10^−5^
3	Low	14	12	6	1 × 10^−5^
4	Low	106	96	48	1 × 10^−5^
5	Low	260	240	120	1 × 10^−5^
6	High	512	450	1	1 × 10^−5^

**Table 4:** Experimental results of the prescreening stage. FoM is the figure of merit.

Image ID	Processing Time	Targets Detected	Real Ship Targets	Correct Targets Detected	False Alarms	Targets Missed	FoM
1	2.7 s	28	19	19	9	0	0.68
2	2.5 s	6	6	6	0	0	1
3	1.4 s	6	6	6	0	0	1
4	0.46 s	4	4	4	0	0	1
5	0.45 s	11	11	11	0	0	1
6	0.67 s	14	12	12	2	0	0.86

**Table 5:** L (length), W (width) and AR (aspect ratio) of targets for test image ID 1 (L and W are measured in pixels).

**Candidate**	**1**	**2**	**3**	**4**	**5**	**6**	**7**	**8**	**9**	**10**	**11**	**12**	**13**	**14**
L	31.6	25.6	22.7	23.5	28.4	17.7	26.6	17.9	29.8	33.4	28.2	28.6	31.4	28.0
W	7.0	9.7	8.2	8.1	8.4	9.5	9.9	9.8	8.1	9.2	10.3	10.1	9.1	8.3
AR	4.5	2.6	2.8	2.9	3.4	1.9	2.7	1.8	3.7	3.6	2.7	2.8	3.5	3.4
**Candidate**	**15**	**16**	**17**	**18**	**19**	**20**	**21**	**22**	**23**	**24**	**25**	**26**	**27**	**28**
L	34.7	21.7	41.2	27.8	14.6	20.8	15.4	14.6	23.0	12.3	51.0	16.7	11.3	16.2
W	9.6	9.6	8.0	10.2	9.2	9.3	9.5	9.1	6.9	11.1	6.8	5.7	8.3	8.4
AR	3.6	2.3	5.2	2.7	1.6	2.2	1.6	1.6	3.3	1.1	7.5	2.9	1.4	1.9

**Table 6:** Preliminary discrimination results and confidence probabilities by CoD (confidence discrimination based on features) for test image ID 1 (√ represents ship target and × represents false alarms, P is the confidence probability).

**Candidate**	**1**	**2**	**3**	**4**	**5**	**6**	**7**	**8**	**9**	**10**	**11**	**12**	**13**	**14**
	**√**	**√**	**√**	**×**	**√**	**×**	**√**	**√**	**√**	**√**	**√**	**√**	**√**	**√**
P	**0.078**	0.52	**0.19**	**0.087**	0.41	**0.23**	**0.14**	**0.084**	0.42	0.49	0.29	0.51	**0.19**	0.46
**Candidate**	**15**	**16**	**17**	**18**	**19**	**20**	**21**	**22**	**23**	**24**	**25**	**26**	**27**	**28**
	**√**	**√**	**√**	**√**	**×**	**√**	**×**	**×**	**×**	**×**	**×**	**×**	**×**	**×**
P	0.51	0.38	0.51	0.51	0.42	0.27	0.48	0.45	0.27	0.51	0.40	0.51	0.51	**0.18**

**Table 7:** Final discrimination results based on CeD (certain discrimination based on features) and CoD for test image ID 1 (√ represents ship target and × represents false alarms, P is the confidence probability).

**Candidate**	**1**	**2**	**3**	**4**	**5**	**6**	**7**	**8**	**9**	**10**	**11**	**12**	**13**	**14**
	**√**	**√**	**√**	**√**	**√**	**√**	**√**	**√**	**√**	**√**	**√**	**√**	**√**	**√**
P	1	0.52	1	1	0.41	1	1	1	0.42	0.49	0.29	0.51	1	0.46
**Candidate**	**15**	**16**	**17**	**18**	**19**	**20**	**21**	**22**	**23**	**24**	**25**	**26**	**27**	**28**
	**√**	**√**	**√**	**√**	**×**	**√**	**×**	**×**	**×**	**×**	**×**	**×**	**×**	**√**
P	0.51	0.38	0.51	0.51	0.42	0.27	0.48	0.45	0.27	0.51	1	0.51	0.51	1
